# Fractionating the unitary notion of dissociation: disembodied but not embodied dissociative experiences are associated with exocentric perspective-taking

**DOI:** 10.3389/fnhum.2013.00719

**Published:** 2013-10-30

**Authors:** Jason J. Braithwaite, Kelly James, Hayley Dewe, Nick Medford, Chie Takahashi, Klaus Kessler

**Affiliations:** ^1^Behavioural Brain Sciences Centre, School of Psychology, University of BirminghamBirmingham, UK; ^2^Sackler Centre for Consciousness Science, University of Sussex, BrightonEast Sussex, UK; ^3^Aston Brain Centre, School of Life and Health Sciences, Aston UniversityBirmingham, UK

**Keywords:** perspective-taking, anomalous bodily experiences, out-of-body experience, dissociation, depersonalization

## Abstract

It has been argued that hallucinations which appear to involve shifts in egocentric perspective (e.g., the out-of-body experience, OBE) reflect specific biases in exocentric perspective-taking processes. Via a newly devised perspective-taking task, we examined whether such biases in perspective-taking were present in relation to specific dissociative anomalous body experiences (ABE) – namely the OBE. Participants also completed the Cambridge Depersonalization Scale (CDS; Sierra and Berrios, 2000) which provided measures of additional embodied ABE (unreality of self) and measures of derealization (unreality of surroundings). There were no reliable differences in the level of ABE, emotional numbing, and anomalies in sensory recall reported between the OBE and control group as measured by the corresponding CDS subscales. In contrast, the OBE group did provide significantly elevated measures of derealization (“alienation from surroundings” CDS subscale) relative to the control group. At the same time we also found that the OBE group was significantly more efficient at completing all aspects of the perspective-taking task relative to controls. Collectively, the current findings support fractionating the typically unitary notion of dissociation by proposing a distinction between *embodied dissociative experiences* and *disembodied dissociative experiences* – with only the latter being associated with exocentric perspective-taking mechanisms. Our findings – obtained with an ecologically valid task and a homogeneous OBE group – also call for a re-evaluation of the relationship between OBEs and perspective-taking in terms of facilitated disembodied experiences.

## INTRODUCTION

Stable self-consciousness, which supports appropriate behavior and experience, is dependent on a legion of multi-sensory co-ordinated processes acting in concert to maintain a coherent sense of the embodied *self* over space and time. These underlying processes include the multi-sensory spatial coding of both one’s own body, the environment, and the constant interactions between body and environment. However, this typically stable process can break down in certain circumstances, leading to striking distortions in body-image and dissociative anomalous body experiences (ABE). One such hallucination that has received growing interest in recent years is the out-of-body experience (OBE).

The OBE can be defined as an experience where the individual “*perceives his/her environment from a perspective outside of their physical body*.” Therefore, a fundamental core aspect to the OBE is the overwhelming sense that one is experiencing the world from and external, exocentric perspective (Eastman, 1962; Green, 1968; Palmer, 1978; Blackmore, 1982; Irwin, 1985). In this sense OBE has been discussed in relation to deliberate processes of egocentric transformation and perspective-taking (e.g., Blanke et al., 2005; Braithwaite and Dent, 2011).

The current and dominant view is that the OBE occurs due to a temporary disruption in multi-sensory integration processes, where stable egocentric processing has become impaired to such an extent that it can no longer represent a coherent sense of embodied “self” (see Blanke and Arzy, 2005; Blanke and Mohr, 2005; Blanke and Metzinger, 2009 for reviews). Although it is not entirely clear how such transient disruptions occur (even more so in non-clinical samples), other independent findings have shown that OBE groups can display; (i) elevated scores on measures of anomalous experience related to disruptions in temporal-lobe processing; (ii) biases in body-transformation/perspective-taking processes; and (iii) elevated signs of visual cortical hyperexcitability – which were absent from both control groups and non-visual hallucination groups (Braithwaite et al., 2011,2013a,b).

In addition, behavioral studies have argued that the brain processes involved in the mental transformation of one’s own body may be the same as those implicated in the computation of an exocentric perspective (for review, see Kessler and Rutherford, 2010; Kessler and Thomson, 2010; Kessler and Wang, 2012; Popescu and Wexler, 2012; van Elk and Blanke, 2013) and particularly in the OBE (Cook and Irwin, 1983; Blackmore, 1987; Brugger, 2002; Blanke and Arzy, 2005; Blanke et al., 2005; Arzy et al., 2006; Mohr et al., 2006; Easton et al., 2009; Overney et al., 2009; Braithwaite et al., 2011). Most of the latter studies used performance at the “own-body-transformation” (OBT) task to explore perspective-taking and have implicated the temporal–parietal junction in the mental transformation of one’s own body and perspective (see Blanke et al., 2005). However, only a handful of these studies actually explored performance on this task in direct relation to samples reporting OBEs – and these have produced diverse results (Easton et al., 2009; Braithwaite et al., 2011).

Interestingly, impairments and not benefits, at OBT tasks have been shown for participants who scored positively on a measure of perceptual aberrations related to schizotypy (Mohr et al., 2006) and more recently for those specifically reporting OBEs (Braithwaite et al., 2011; though see also Easton et al., 2009). These tasks present observers with a schematic figure which is either facing the observer or facing away from the observer. Participants are instructed to try to adopt the perspective of the figure and hence engage perspective-taking processes and decide on what hand (left/right) the figure wearing a distinctive glove and bracelet.

Although these tasks were originally thought to measure similar perspective-taking mechanisms to that implicated in the out-of-body perspective, findings where schizotypes and OBE groups were impaired at the task, appear to go against the intuitive idea that those reporting dissociative experience should be better at exocentric perspective-taking. Whether the typical OBT task truly is an exocentric perspective-taking task has now been questioned on the grounds that with only two exemplar avatars, other rule-based contingency strategies may be impacting more on performance rather than exocentric perspective-taking (Braithwaite and Dent, 2011; Gardner and Potts, 2011; Gronholm et al., 2012; Kessler and Wang, 2012; May and Wendt, 2012; see also Pezzulo et al., 2013).

Collectively, the evidence for clear benefits in perspective-taking, for those individuals prone to anomalous disembodied and dissociative experiences, is currently unclear, contentious, and awaiting clarification. This is likely due, in part, to; (i) diverse methodologies used to examine such processes; (ii) not all previous studies claiming to explore the mechanisms of OBEs have actually used OBE samples and; (iii) the use of other distinct groups of hallucinators (e.g., schizotypes) that may themselves reflect quite different underlying mechanisms that do not include exocentric hallucinations. These different mechanisms may well be masked as they currently exist under the generic umbrella concept of “*dissociative experience*” not all of which would conceivably index exocentric processes. As a consequence it becomes important to examine the OBE not just in its own right, but alongside other similar though distinct dissociative experiences.

Shedding light on this currently ambiguous situation will also help our understanding of the embodied processes involved in more deliberate forms of perspective-taking, where the social and/or spatial goals might be conscious and deliberately chosen, yet, where the actual mechanism for transforming the “ego” into an exocentric perspective seems to be strongly embodied (Kessler and Thomson, 2010) and compulsory rather than deliberately chosen (Kessler and Wang, 2012), and might therefore strongly resemble the spontaneous OBT underlying OBE.

### DEPERSONALIZATION, DEREALIZATION, AND THE OBE

Early accounts for the OBE came from psychiatry, where it was cast as a specific instance of depersonalization (Noyes and Kletti, 1976, 1977). Depersonalization disorder (DPD) is a syndrome which reflects a severe disruption in self-awareness that can include dissociative experiences (Sierra and David, 2011). Depersonalization itself typically refers to an unreality of the self. Patients classically describe feelings of remoteness, estrangement from the self, feeling like a robot or automaton, and a flattening of emotional affect (Sierra, 2009; Sierra and David, 2011). The related concept of derealization (DR) which can commonly co-occur with depersonalization, refers more to an unreality of surroundings – where patients typically describe experiencing the world through a fog, a veil, a bubble and being “detached” from their surroundings (Sierra and David, 2011).

The relationship between OBEs and DPD-DR has been questioned. For example, in the OBE the experience is often described as being extremely vivid, convincing, striking, and very real. Individuals often describe a heightened sense of awareness and increased clarity of thought during the experience (see Blackmore, 1982). In contrast, DPD-DR experiences are often described as having a dulled or flattened affect, loss of emotional coloring, and can be somewhat dreamlike (Gabbard et al., 1981, 1982; Twemlow et al., 1982). In addition, DPD-DR experiences typically occur in stressful situations, whereas the OBE can equally occur spontaneously in quite relaxed conditions. These phenomenological and contextual differences have led to the view that OBEs and the ABE reported in DPD-DR are not the same and may reflect quite different neurocognitive underpinnings (Gabbard et al., 1981, 1982; Blackmore, 1982; Twemlow et al., 1982; Gabbard and Twemlow, 1984, 1986; see also Sierra, 2009 for a discussion).

There is some confusion over the terminology used when describing the anomalous experiences reported by DPD-DR patients that may contribute to continued misunderstandings about the prevalence of OBEs in DPD-DR as well as the clinical construct of DPD-DR itself (see Sierra and Berrios, 1997; Medford et al., 2005 for detailed discussions). For example, while some experiences might be described as “disembodied” or “dissociative” OBEs themselves are rarely, if ever, reported by patients with DPD-DR. What patients appear to be describing is that they feel their bodies are unreal and do not belong to them. However, a closer examination of these accounts shows that the perceiving “self” is still typically described as being located inside the physical self – so there is no external “disembodiment” or shift in experiential perspective. The term “disembodiment” and perhaps to a lesser extent “dissociation” can be taken to imply that DPD-DR experiences commonly involve experiences where the perceiving “self” shifts perspective from an egocentric and embodied one, to an exocentric and disembodied one (an OBE). However, for DPD-DR this is rare, so much so that some have noted the complete absence of OBEs in DPD-DR patient populations (Sierra, 2009).

### OVERVIEW OF THE CURRENT STUDY

The present study sought to examine cognitive biases in perspective-taking/body-transformation processes that may be implicated in predisposition to hallucinatory experiences that involve a shift in self-perspective (the OBE). If the striking phenomenological aspects of OBEs are based in some form of involuntary exocentric perspective-taking, then individuals prone to OBEs may also display distinct performance in a deliberate perspective-taking task. An intuitive prediction is that those prone to OBEs would be better at a perspective-taking as they may recruit the same transformational mechanisms underlying the OBE. Although some previous research has shown the opposite pattern, where OBE groups have shown impaired performance (Braithwaite et al., 2011), the actual task employed in these studies has been questioned (Braithwaite and Dent, 2011; Braithwaite et al., 2011).

**FIGURE 1:**
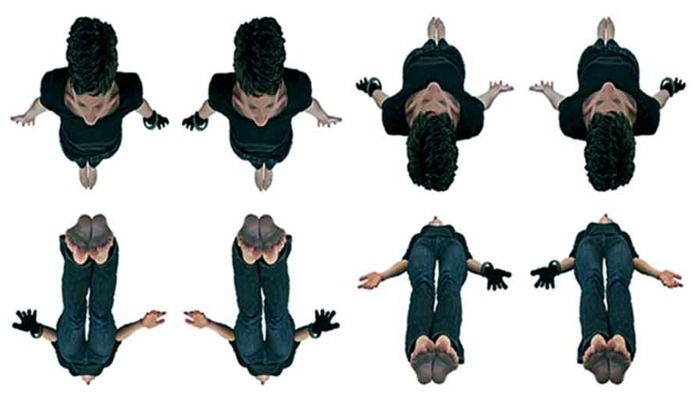
**Stimuli from the new Human-own-body-transformation task (HOBT) where avatars from both ‘Above’ (top row) and ‘Below’ (bottom row) perspectives were used**. Note, while all avatars require the transformation of plane, the two avatars in the upper row/ left hand side, and lower row right-hand side also require a rotation of body as well. Therefore the stimuli are distinguishable along two a-priori dimensions, one of plane and one of body-rotation.

Therefore, a new perspective-taking task was devised for this study, where a human female avatar could be viewed from either an “Above” viewpoint (above the head of the avatar) or “Below” viewpoint (below the feet of the avatar). Thus, unlike many previous studies, here the avatar was rotated around the horizontal axis and not the more typical vertical axis (or what some describe as around the sagittal plane and not around the transverse plane; Carpenter and Proffitt, 2001; Creem et al., 2001). In addition to any transformation of plane/viewpoint required, half of the stimuli also required a (mental) rotation of the participant’s body in order to fully transform and match their perspective to that of the avatar (see **Figure 1**).

There were two advantages from these new manipulations. Firstly, these manipulations produced eight separate avatars, four from the above viewpoint and four from the below viewpoint, but two of these four also differed in terms of requiring body-rotation. Previous OBT tasks have typically used only exemplars with two different body positions (e.g., facing/behind). As a consequence the current study is arguably more resistant to the emergence of non-spatial basic contingency-based or rule-based strategies emerging across trials.

Secondly, the use of “Above” viewpoints is more phenomenologically similar to the perspective reported in many visual OBEs (see also Schwabe et al., 2009). As a consequence, the current perspective-taking transformations are more in line with those implied in accounts of OBEs. Finally, the presence of both a transformation of plane and body-rotation facilitates a separate exploration of these factors in relation to overall body transformations, perspective-taking and spatial processing in relation to OBEs.

In addition to the new behavioral tasks, all participants were measured for their proneness to dissociative anomalous experiences via the administration of the Cambridge Depersonalization Scale (CDS; Sierra and Berrios, 2000), which contains measures of both ABE and anomalous experiences of one’s surroundings (derealization). As noted in the Introduction, there has been some debate as to the relationship between depersonalization and OBEs (Noyes and Kletti, 1976, 1977; Gabbard et al., 1981, 1982; Blackmore, 1982; Gabbard and Twemlow, 1984; see also Sierra, 2009). However, there have been few, if any, experimental investigations of these factors together. Importantly, the ABE measured by the CDS are more related to embodied anomalous experiences, where the self remains within the body and is not transposed into an exocentric perspective. It is not at all clear whether OBE groups also experience elevated levels of these potentially related experiences or whether the OBE tends to occur in isolation to these other experiences. In addition, the CDS also contains a measure of derealization, where individuals report being cut-off and alienated from their surroundings. In light of recent accounts from cognitive neuroscience on the role of a breakdown in multi-sensory integration underlying the OBE, any depletion or disruption in incoming sensory signals from the outside world may act to destabilize internal models of the bodily self. As a consequence, the OBE group may well display elevated signs of derealization, even more so, than the embodied ABE associated with depersonalization *per se*.

## MATERIALS AND METHODS

### PARTICIPANTS

Sixty-two participants took part in the present study. Of these, 47 (82%) were female and 60 (96%) reported that they were right-handed. None reported any personal medical history of seizure, epilepsy or were diagnosed as having migraine. All participants were undergraduate or postgraduate students (MSc/PhD) from the School of Psychology at the University of Birmingham, UK. Participants ranged in age from 18 to 28 years (average age of 21.5 years). All received course credit for taking part in the study.

### QUESTIONNAIRE MEASURES

#### The Cambridge Depersonalization Scale

The CDS (Sierra and Berrios, 2000) is a 29-item psychometrically established measure of dissociative anomalous experiences associated with the construct of depersonalization (anomalous experiences of the “self”) and derealization (anomalous experiences of ones surroundings). Two responses to each question are given on 5-point Likert scales, one response for “Frequency” and one for “Duration” and the final score for any item is the summed output of both these responses (giving a potential range of scores between 0 and 290).

It is now recognized that clinically significant depersonalization–derealization (DPD-DR) is best considered as a syndrome rather than a single phenomenon (Sierra, 2009), since it involves alterations in the quality of subjective experience across a range of different experiential domains (see, for example, Medford et al., 2005). Although this multi-factorial understanding of DPD-DR has been present in descriptive literature for many decades (see also Ackner, 1954; Sierra and Berrios, 1997) it is only recently that it has been confirmed by empirical phenomenological studies (Sierra et al., 2005; Simeon et al., 2008) which have examined the clustering of CDS items into different factors. In the study by Sierra et al. (2005), CDS items were shown to segregate into four distinct factors, which the authors termed (i) ABE; (ii) emotional numbing (EN, analogous to the term “de-affectualization”), (iii) anomalous sensory recall (ASR), and (iv) alienation from surroundings (AFS or derealization; Sierra et al., 2005). Previous research on patients has shown that a cut-off point of 70 yields a sensitivity of 75% (specificity of 87%) and has high internal consistency (Cronbach’s alpha = 0.89) and half-split reliability (0.92; see Sierra and Berrios, 2000; Sierra, 2009). Importantly, it should be noted that there is no explicit question on the CDS for OBEs. The ABE questions typically describe anomalous states that are more associated with embodied perceptions (see General Discussion for further elaborations)^1^.

#### The OBE pre-screen

A pre-screen questionnaire to establish the presence of OBEs and some basic phenomenological information about them was also administered. This questionnaire has been used and detailed in previous studies from our laboratory (Braithwaite et al., 2011, 2013a,b). Participants are initially asked the question: “*Have you ever had an experience where you have perceived/experienced the world from a vantage point outside of the physical body?*” In addition to this question participants were given further qualifying information that (i) such an experience can feel totally real at the time of the experience with all the phenomenological qualities of veridical perception and (ii) that such experiences can be fleeting and transient or more sustained. If a response of “yes” was provided then additional contextual and situational information about the experience(s) was also ascertained such as how often they had experienced an OBE, whether the experience was visual in nature, whether they saw their physical self during the experience, and the perspective from which they experienced the world or self (above, below, in front, behind, laterally, or other). Associated phenomenology was also documented (e.g., feelings of dizziness, floating sensations, disorientation, dissociation, duality of consciousness, other sensory experiences, etc). This questionnaire also allowed us to ensure the participant themselves had not incorrectly defined their own experiences as OBEs, when in fact they might not be consistent with classical definitions.

### PROCEDURE AND STIMULI: PERSPECTIVE-TAKING TASK

All participants took part in a newly devised version of a perspective-taking task, which for clarity and conciseness we now refer to as the Human OBT (HOBT) task. Unlike previous versions of the OBT task, the present stimuli consisted of both aerial (elevated/above the avatars head) and low (beneath the avatars feet) color photographic views of a human female avatar. In each photograph, the avatar was wearing a distinctive glove/bracelet on one hand. The avatar could be facing in two directions (toward the top or bottom of the screen), from either the elevated or beneath viewpoints, thus generating eight possible exemplar photographs (four from each viewpoint) when combined with the differing hands wearing the glove/bracelet. To successfully solve the task the avatars differed on two main *a priori* dimensions.

For example, all avatars required a transformation of plane where the viewpoint of the participant or the avatar itself could be transformed. In addition, some of the avatars (see **Figure 1**) also required an additional step of mental body-rotation in order to match the perspectives between participant and avatar. The *a priori* prediction was that reaction times (RTs) for those avatars requiring the additional step would be increased. These stimuli were presented centrally, at fixation, against a white background on an 17-inch Samsung CRT monitor coupled to a Pentium PC. The stimuli are shown in **Figure 1**. The experiment was programed in E-prime software v2.1 (Psychology Software Tools).

The stimuli were viewed at an unfixed but general distance of 60 cm and were approximately 110 mm wide × 75 mm high. Each trial began with the presentation of a black central fixation cross on a white background. The fixation cross was presented for 1000 ms followed by the presentation of the human avatar which remained on the screen until response. There was an inter-stimulus interval of 1000 ms between trials.

All stimuli were presented within one single block of 96 trials (48 per perspective). Participants were instructed to imagine themselves to be in the figure’s body position and to adopt the appropriate perspective of the figure. Once done, they had to respond to whether the glove was on the left hand (up-arrow keyboard response) or right hand (down-arrow keyboard response) of the human avatar. The presentation of the different stimuli was randomized within the experimental block of trials. The experiment began with a separate block of 16 practice trials which were not analyzed but used so that participants could learn the correct response-mapping. Participants were instructed to respond as fast and as accurately as they could. The experiment lasted approximately 40 min (including the administration of the questionnaires). The questionnaires were always completed after the perspective-taking task.

## RESULTS

For the perspective-taking task, RTs were made fit for analysis in the following way. Firstly, all incorrect responses were identified and removed from the analysis. This revealed an overall response accuracy rate of 94%. Secondly, all outliers (deemed at ±2.5 standard deviations from the mean) and responses faster than 200 ms were also discarded. Any participant with less than 80% accuracy at the task was also removed from the analysis. This procedure led to five participants being removed from the analysis^2^. The following analysis was carried out on the remaining sample of 57 participants. An overall measure of performance was calculated where the proportion of correct responses was divided into the RTs providing a measure of efficiency (Townsend and Ashby, 1983; see also Rach et al., 2011). All statistical tests are reported two-tailed and, where necessary, *p*-values have been corrected for multiple comparisons (via the Bonferroni procedure) and corrected degrees of freedom are reported if non-homogenous variability occurred.

Of the remaining participants, 17 (30%) claimed to have experienced an OBE at some point in their life. The remaining 70% made up the non-OBE control group. The OBE pre-screen questionnaire revealed that the entire OBE group reported their experiences had a strong visual component to them, where they experienced themselves or their local environment from an external and exocentric perspective. In addition, all reported an elevated perspective to their experiences, as if they were looking down on the world and/or themselves. Although other multi-sensory information was also noted and contributed to the realism of the experience (e.g., vestibular distortions/floating sensations) in all cases these always co-occurred with visual aspects of the experience.

### CAMBRIDGE DEPERSONALIZATION SCALE

Overall summed scores were explored for normality via a Shapiro–Wilk test and were found to be borderline non-normally distributed [*W* = 0.96 (df = 57), *p* < 0.05]. As a consequence, these questionnaire data were explored with non-parametric statistics. The overall sample mean score for the CDS was $\bar{X}$ = 30.5 (median = 29.3, and range = 0–84). Two participants scored just above the score of 70 (scores of 71, 84) and one was borderline (score of 66).

**FIGURE 2:**
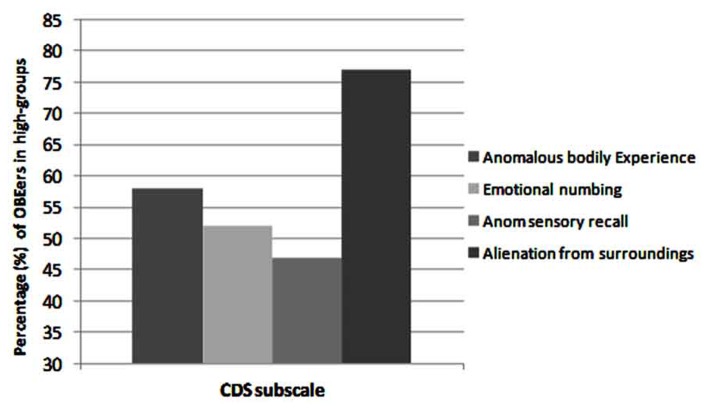
**Descriptive statistics (percentages) of those reporting OBEs in the high-groups of each subscale from the Cambridge Depersonalization Scale (CDS)**.

A median-split analysis was carried out independently on all four subscales of the CDS and the percentage of those reporting OBEs occurring in the high-groups of these subscales was calculated (see **Figure 2**). This revealed that the high-ABE and AFS groups contained the largest numbers of those reporting OBEs. Interestingly, these descriptive statistics show that 77% of those reporting OBEs placed in the high-AFS group (i.e., increased signs of derealization).

**FIGURE 3:**
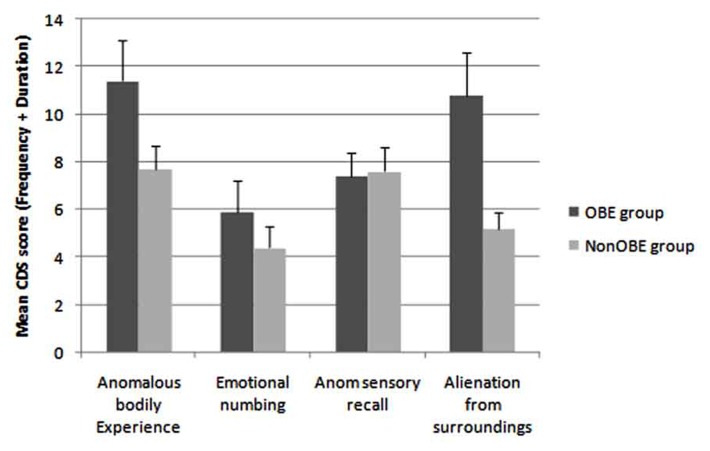
**Mean CDS scores for each of the 4 subscales (identified by Sierra and Berrios, 2000) plotted for both the OBE and non-OBE control groups (error bars = 1 SE)**.

The mean CDS scores for all subscales and for both the OBE group and non-OBE controls are graphically represented in **Figure 3**. These differences were formally compared by a series of Mann–Whitney *U*-tests. Although the largest effects appear to be present for both ABE and AFS measures, after correction for multiple comparisons, only the difference between the groups for the AFS subscale was significant (*U* = 176.00, *Z* = -2.88, *p* < 0.005). The OBE group produced significantly higher scores on measures of AFS ($\bar{X}$ = 10.8, SE = 1.6) than the control non-OBE group ($\bar{X}$ = 5.2, SE = 1.2; see **Figure 3**). Although this general pattern also held for measures of ABE (OBE $\bar{X}$ = 11.8, SE = 1.8; non-OBE control $\bar{X}$ = 7.5, SE = 0.08), this was not reliable after correction for multiple comparisons (*U* = 233.50, *Z* = -1.86, *p* = 0.08).

Seventy-seven percent of those claiming OBEs in the present sample placed in the high-AFS group (suggesting that the majority of this group displayed elevated signs of derealization experiences). In addition, the OBE group reported significantly higher degrees of AFS relative to the non-OBE control group. The effect for the OBE group to display increased scores on measures of ABE, though showing signs of being present, failed to be reliable. No other factors reliably distinguished the groups.

### PERFORMANCE AT THE HOBT TASK

**FIGURE 4:**
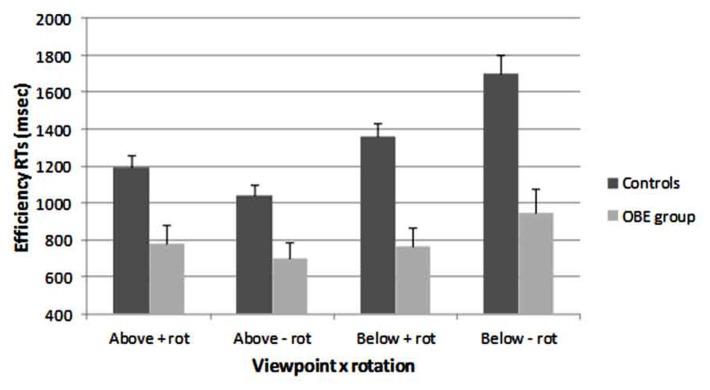
**Efficiency RTs for the HOBT task plotted for both Groups (Controls and OBE group) both perspectives (Above and Below) and whether an additional body-rotation was required or not (+ rot = requires body rotation / - rot = does not require body rotation: error bars = 1 SE)**.

Mean correct efficiency RTs for the HOBT task are plotted in **Figure 4**. Performance at the HOBT task was examined by a 2 (Group: Controls vs OBE group) × 2 (Viewpoint Above/Below) × 2 (Body rotation: Rotation vs No rotation) mixed ANOVA applied to the efficiency RTs. The main effect of Group was significant, *F*(1, 55) = 24.33, *p* < 0.001; as was the main effect of Viewpoint, *F*(1,55) = 30.70, *p* < 0.001. On the whole, the OBE group was significantly more efficient ($\bar{X}$_diff_ = 528 ms) than the non-OBE control group at the HOBT task. In addition, both groups were significantly more efficient overall at Above viewpoints, relative to below viewpoint ($\bar{X}$_diff_ = 264 ms). In contrast, the main effect of Rotation was not significant, *F*(1,55) = 1.67 *p* = 0.202 ($\bar{X}$_diff_ = 73 ms). The Viewpoint × Group and the Viewpoint × Rotation interactions were significant, *F*(1,55) = 10.04, *p* < 0.005; and *F*(1,55) = 15.64, *p* < 0.001, respectively. However, the Rotation × Group interaction was not significant, *F*(1,55) = 0.178, *p* = 0.674. Finally, the three-way interaction between Group × Viewpoint × Rotation was not significant, *F*(1, 55) = 1.40, *p* = 0.242.

The significant interactions were explored further via a series of within subjects *t*-tests carried out separately for each group, for each viewpoint and rotation condition. These data are given in **Table 1**.

**Table 1:** Breakdown of the separate effects of Viewpoint and Rotation within the two groups.

Condition	$\bar{X}$_diff_ (ms)	*t*-Statistic	df	*p*-Value
Controls: Above rot – No rot	146	2.79	39	<0.01*
Controls: Below rot – No rot	-339	2.80	39	<0.01*
OBE group: Above rot – No rot	82	1.91	16	=0.07
OBE group: Below rot – No rot	-180	3.42	16	<0.005*

* = results are significant.

To explore the overall cost of viewpoint between the groups, the overall RTs from the “Above” viewpoint were subtracted from RTs from the “Below” viewpoint for both the OBE and control groups. This generated two sets of difference scores. These differences were explored via a between-subjects *t*-test which was significant [*t*(52.9) = 4.39, *p* < 0.001]. On the whole, non-OBE controls were more impaired (by 298 ms) by the cost for below viewpoints than the OBE group.

To summarize, both groups were more efficient at the Above viewpoint compared to the Below viewpoint. In addition, the OBE group was significantly more efficient at all aspects of the HOBT task relative to the non-OBE control group. For Above viewpoints, there was a general trend for a cost to efficiency if an additional body-rotation was required (in addition to any transformation of plane) though this was only reliable for the control group. The pattern of findings for Below viewpoints was reversed with, rather surprisingly, efficiency being improved for those avatars that might require an additional step of body-rotation as well as any transformation of plane. These findings are discussed more fully in Section “General Discussion.”

## GENERAL DISCUSSION

The present study examined biases in exocentric perspective-taking/body-transformation processes in relation to predisposition to hallucinatory experiences that involve a shift in self-perspective – the OBE. In addition, signs of embodied anomalous experiences associated with depersonalization/derealization (DPD-DR), with OBE groups, were also explored.

The OBE is, by definition, an anomalous experience revolving around a shift in the perspective of the experiencing self “outside of his/her body.” In line with previous research (Blanke et al., 2005; Braithwaite et al., 2011), a premise of the present study was that if OBEs are based in some form of disruption in the mechanisms underlying stable egocentric processing and/or the efficient use of exocentric perspective-taking processes, then these individuals may display distinct performance at a task which is sensitive to these processes. In addition to this, we also examined the rate and range of other dissociative anomalous experiences to explore their association with the OBE and exocentric perspective-taking.

There was a borderline significant trend for the OBE group to report more additional ABE relative to control groups. This observation for a general trend of elevated egocentric ABEs (associated with depersonalization) for the OBE group is new, though complements other research showing increased somatoform distortions for these groups (Irwin, 2000; Murray and Fox, 2005). Both the ABE subscale in the present study, and the somatoform dissociation scale used by previous studies, include only items related to either altered bodily sensations, or egocentric dissociative experiences. Clearly, the OBE is a specific form of exocentric ABE and can co-occur with other egocentric dissociative phenomenology. The weaker effects seen here for the ABE subscale are possibly due to the fact that this is a small subscale of items (much smaller than the full measures used in previous studies), containing items more focused on dissociative experiences, rather than specific somatoform distortions (though the two can be related). 

In contrast to the pattern seen for all other subscales (ABE, EN, ASR), the OBE group did provide clear and significantly elevated scores on measures of AFS (derealization) compared to the non-OBE control group. Indeed, an exploratory median-split analysis carried out on the whole sample revealed that 77% of the OBE group fell in the high-scoring group for derealization. The relationship between derealization and the OBE is both new and interesting as it might imply that the OBE itself is a response to a temporary lack of connection between the “self,” and the surrounding world.

By this account, the specific neurocognitive biases underlying derealization may increase the disconnection between the bodily self and one’s own surroundings to such an extent that internal representations of the body/self become unstable or degraded in some way. At the very least, incoming sensory information may become ambiguous under conditions of increased derealization. The net consequence of this is that typically stable egocentric representations of the self might become so disrupted that they can no longer support coherent embodied conscious experience. Under some circumstances this might simply result in the dissociative anomalous experiences reported by DPD-DR patients and their non-clinical counterparts (e.g., estrangement from the self, bodies feeling unreal, surroundings feel dreamlike, dull, and deadened). However, in other instances these situations may act as a catalyst for OBEs providing the individual also displays additional cognitive biases in exocentric perspective-taking. This in itself is noteworthy and has implications for the broader debate on whether the OBE is or is not related to DPD-DR (see Sierra et al., 2002; Sierra, 2009; Sierra and David, 2011).

The observation that the OBE group were also significantly more efficient at the objective HOBT perspective-taking task relative to the non-OBE control group is particularly noteworthy. This was the case across all viewpoints and body-rotation permutations of the stimuli. Both groups found the Above viewpoints easier than the Below viewpoints (see also Schwabe et al., 2009 for similar findings with only control groups). This is to be expected and likely reflects both a greater familiarity with seeing bodies from elevated/above viewpoints and also the clear view of the head/shoulder region may act as a useful anchor point (e.g., Kessler and Rutherford, 2010), with which to carry out the transformations necessary to complete the task efficiently and successfully.

An unexpected result was the diverse role of the “Rotation” factor across the different viewpoints. For Above views, there was a general cost to efficiency if both a transformation of the body and plane was required. This cost was significant for control groups, and borderline reliable for the OBE group (see **Table 1**). This overall finding is in line with our *a priori* intuitive prediction that avatars involving two separate transformations (of both plane and body) will be less efficient than those avatars only involving one transformation. The exact opposite pattern occurred for below viewpoints, where RTs were generally increased, but where efficiency was actually benefited by the apparent needs of both a transformation of plane and body-rotation and hampered where apparently only one transformation was required. This result is supported further in that it was observed for both the OBE and control groups.

One possible explanation is that for the “Below - Rotation” condition, and this condition alone, participants may not be carrying out the spatial transformation in a similar manner to the other instances. For example, for both “Above” viewpoints, a clear and familiar view is provided and a salient anchor point (i.e., the head) contribute to the transformations required to efficiently solve the task at hand. Here, either a transformation of plane, of body-rotation, or both are required. It is also, due to the familiar perspective, quite salient which of these processes are best suited to the situation.

However, the “Below - Rotation” condition, presents a view of an avatar which we rarely, if ever experience in daily life: it would require us either looking up at people through a glass floor, or watching superman flying over our heads. In contrast, the “Below + Rotation” condition is identical to a person lying on a bed, thus, a quite familiar view. We therefore suggest that in the “Below - Rotation” condition, instead of a simple transformation of plane, participants may first rotate the whole avatar (like hands rotating on a clock face), in order to place the head toward the top, but in so doing, this now generates the need for an additional body-rotation. Therefore this condition may actually elicit two rotational strategies rather than our assumed one transformation – thus impacting on the efficiency of performance. As suggested, this may be due to the absence of a salient anchor point and unusual view of the human body with which to assign the appropriate initial transformation (e.g., Grabowski, 1999; Kessler and Rutherford, 2010).

### EMBODIED AND DISEMBODIED DISSOCIATIVE ANOMALOUS EXPERIENCES

The present study provides preliminary evidence for fractionating the unitary notion of “dissociation” underlying ABE. We suggest that one important factor for consideration when examining the mechanisms underlying dissociative states is whether the dissociation being examined is from an egocentric or “embodied” perspective or whether it is from an exocentric or “disembodied” perspective (or indeed both; e.g., as in cases of heautoscopy; Brugger et al., 1997; Brugger, 2002). As a consequence it might be helpful to conceptually view the legion of dissociative states of the self as being representative of either “*embodied dissociation*” (e.g., dissociative experiences reported in depersonalization, schizophrenic loss of body boundaries, autoscopy, sensed-presence experiences) where the perceiving “self” remains firmly located within the physical body, or “*disembodied dissociation*” (i.e., OBEs) where the perceiving self appears liberated from its egocentric physical moorings. Only the latter implies a bias for additional exocentric perspective-taking processes underlying the phenomenology of the anomalous experience^3^,^4^.

Although speculative, this view is supported by findings from the present study as well as the broader literature. The crucial and major difference between the groups in the current investigation appears to have been the presence of exocentric OBEs, which may have resulted from the co-presence of elevated signs of derealization and biases in exocentric perspective-taking processes. It was clearly the case that the OBE group experienced other forms of egocentric ABEs, but the presence of these additional egocentric ABEs did not appear to be as strongly related to performance at the exocentric perspective-taking task.

Therefore, although the OBE group was a group which reported additional non-exocentric ABEs, performance at the HOBT task appeared to be related more to the co-presence of *disembodied dissociative* experiences that may well have been reliant on an exocentric representation of the self in space (the OBE). The control group, by definition, did not report any instances of *disembodied dissociative* experiences. In addition, their performance at the exocentric perspective-taking task was significantly less efficient than that of the OBE group.

Interestingly, Sierra (2009) notes that while the concept of disembodiment does imply an experience where the “self” is localized outside one’s physical body (analogous to the OBE), in cases of depersonalization, disembodiment is certainly not taken to imply a shift in perspective of the experiencing self at all. Instead, with depersonalization, patients describe “*not really being there*” in an egocentric sense – but do not claim to occupy any external perspective. This supports our argument here that terms like disembodiment and dissociation require a more considered usage when examining cases of OBEs relative to seemingly similar situations like DPD-DR. It would appear that there has been some equivocation over the use of terms like disembodiment over the years which, in no small way, has contributed to confusion over depersonalization and other ABE.

Sierra’s (2009) salient observation shows that the term “*disembodiment*” has often been taken to describe both; (i) what is, in reality, a reduction in saliency of the embodied sense of self – where one is still embodied (egocentrically), but this is greatly weakened/diluted as well as; (ii) being completely disembodied (exocentrically) into another spatial location (the OBE). Because both these factors can occur together and can be dissociated, we recommend abandoning using the term disembodiment for both cases and those representing the former situation.

The revised taxonomy argued for here would help navigate around such confusion, as the concept of disembodiment would only be used for instances where exocentric perspectives are experienced and dominate consciousness. As a consequence of this redefinition, ABEs described by patients with DPD-DR would not be defined as disembodied – though they are clearly dissociative. In other words, one can be dissociated from the self (i.e., estranged from the self) while not necessarily being disembodied from the self.

One argument against this position might be instances where patients may describe no salient experiential perspective, and instances of heautoscopy – where dual egocentric and exocentric perspectives appear to co-exist, are thus not easily accommodated within this re-description. However, our conception is supported by a clear division in empirical performance at a more objective behavioral task, and not just subjective reports in interviews or via questionnaire measures. In addition, the proposed conception does help to; (i) differentiate many dissociative experiences from a variety of neurological, clinical, and psychotic conditions; (ii) adds clarity to the confusion surrounding the nature of ABE in depersonalization; (iii) more clearly highlights the important differences between ABE in depersonalization and the OBE, and; (iv) implicates the possible presence or absence of certain neural networks (exocentric perspective-taking/self-perspective inhibition). Furthermore, identifying experiences that lie outside of these boundaries is still helpful for the development of scientific theory.

In terms of the actual mechanisms mediating the increased efficiency seen for participants predisposed to OBEs, one may think of these simply as an increased ability in exocentric perspective-taking *per se* (i.e., the ability to simply adopt an external point of view). However although intellectually seductive, to some extent these findings may also index a greater ability to suppress the egocentric point of view. There is growing evidence for the existence of both mechanisms of self-perspective inhibition (Vorauer and Ross, 1999; Ruby and Decety, 2004; Samson et al., 2005; van der Meer et al., 2011) and the excitation of exocentric perspectives (Ruby and Decety, 2001; Saxe et al., 2006; Lambrey et al., 2008; see also Zacks et al., 1999, 2000). These may work in concert to achieve exocentric representations underlying striking and convincing multi-sensory hallucinations of the self like the OBE. Both processes may also enjoy diverse neurocognitive underpinnings. One prediction here is that self-perspective inhibition may not, on its own, be sufficient for an OBE to occur. Under these circumstances, individuals may simply report embodied dissociative experiences (e.g., estrangement from the self or “*not being there*”). The *disembodied dissociative* experiences reported by those having OBEs may require additional, alternative and exocentric representations of the self in space.

Interestingly, uniting these themes into a coherent and more comprehensive account of dissociative experiences might also help illuminate theories of both depersonalization and OBEs. For example, as dissociative ABEs reported in depersonalization appear to be entrenched in egocentric/embodied representations, they might reflect an increased and aberrant weighting of internal bodily experiences (perhaps in an attempt to re-establish the egocentric self which is disintegrating). This aberrant weighting or attentional-shift directed toward internal bodily sensations may itself increase the saliency of internal and interoceptive body-sensations and thus contribute to some of the embodied ABEs reported by DPD-DR patients. This would also be consistent with the observation that clinical cases of DPD-DR have identified the presence of hyperreflexivity – where some patients can become obsessive and display an aberrant focus on bodily sensations (Medford et al., 2005; Sierra, 2009; Sass et al., 2013). Similar observations have been made in studies showing that OBE groups can also display increased signs of somatoform dissociation/distortion, revolving around a heightened and magnified sense of self and self-consciousness (Murray and Fox, 2005).

Such a shift to internal representations might also contribute to altered experiences of one’s own surroundings, as attention and processing would be drawn away from processing salient external signals. Conceivably this might contribute, in part, to the nature of the particular phenomenological characteristics of derealization experiences (e.g., observers feel cut-off/detached from the world). If the observer does not have access to additional biases in exocentric representational systems, then they remain embodied, but dissociated and depersonalized. However, in other cases where aberrant activation in exocentric representations also contribute to the experience, which are also temporarily more stable than disrupted egocentric and embodied representations, then an OBE might be more likely to develop.

The present findings may also speak in some way to the ongoing debate over the concepts of depersonalization and derealization – where it has been argued that “pure” cases of derealization are rare in the clinical literature and thus it may not actually reflect a separate construct (see Sierra et al., 2002). Although our present findings are based only on two of the four measures from the CDS, the current findings do imply a stronger effect for derealization (relative to the ABEs associated more with the construct of depersonalization) in relation to OBEs. This provides some tentative support for the view that derealization experiences may well reflect distinct underlying mechanisms, at least for non-clinical hallucinators.

### REMAINING ISSUES

Although there are many variants of perspective-taking tasks in the literature, it is not always clear-cut that the processes required to complete them successfully necessarily recruit exocentric perspective-taking. For example, Braithwaite and Dent (2011) were the first to have questioned these assumptions in relation to the evidence recruited for the standard OBT task used by Blanke and colleagues to examine disruptions in body-transformation/perspective-taking processes (e.g., Blanke et al., 2005; Arzy et al., 2006; Mohr et al., 2006; Easton et al., 2009). One limitation with these earlier incarnations of the OBT task is that it typically recruited only two perspectives in the exemplar stimuli and alternative strategies could easily be developed and used within a block of trials (see also Gardner and Potts, 2011; Gronholm et al., 2012; Kessler and Wang, 2012; May and Wendt, 2012; see also Pezzulo et al., 2013). Although often empirical, it is important to remain aware of the different transformational processes (e.g., perspective-taking, object-rotation, if/then strategies) that may be apparent in a given task (Hegarty and Waller, 2004). 

These limitations should also be considered in relation to the current task. In the context of the current debate it is important to ask if; (i) the tasks used can be reasonably assumed to measure rotational processes (either exocentric perspective-taking, or mental rotation); and (ii) that these particular mechanisms are functionally implicated in disembodied dissociative experiences (i.e., the OBE). Although always problematic to separate, some of the current findings do suggest that rotational/transformational processes, more than alternative non-transformational ones, are indeed playing a role in the current task.

For example, the main effects of Viewpoint, the Viewpoint × Rotation, and Viewpoint × Group interactions would not be expected from some basic form of if/then rule or similar trial-by-trial strategy. These components should be irrelevant to such rule-based strategies. The current HOBT task used two different body positions, from two different viewpoints, and not just a binary viewpoint manipulation (as has been the case with a number of studies; Blanke et al., 2005; Arzy et al., 2006; Mohr et al., 2006; Easton et al., 2009). So the development of alternative strategies, while not impossible, would have to cope with much greater trial-by-trial unpredictability, impairing basic contingency-based and rule-based strategies. In addition, it is not at all clear how or why such contingency-based strategies could explain the effects of Group also seen in the present findings – unless it is argued that such non-spatial strategies relate to the mechanisms underlying the exocentric OBE in some meaningful way.

Furthermore, previous independent investigations that have used the standard OBT task have reported significant costs to RT performance, not benefits, for both OBE samples (Braithwaite et al., 2011) and those showing elevated signs of perceptual aberrations linked to schizotypy (Mohr et al., 2006). This is in contrast to the large and significant improvements to task efficiency found in the present study. Collectively, these findings suggest that the present HOBT task is both methodologically improved and not equivalent to the performance reported for the more traditional version of the task.

Whether the current task predominantly recruits object-rotation or exocentric perspective-taking in the form of mental self-rotation (e.g., Kessler and Thomson, 2010) remains to be explored with future experimentation. In fact, different conditions of the HOBT task may have triggered different strategies of object- vs. self-rotation. We have argued that the condition with the longest RTs, the “Below - Rotation” condition, may have required an initial rotation of the avatar into a more familiar orientation, which is an example of mental object-rotation, while the subsequent steps in this condition as well as the default transformations in the other three conditions may have consisted in mental self-rotation. This is clearly speculative but could be resolved in future studies making use of posture manipulations. Kessler and colleagues (Kessler and Rutherford, 2010; Kessler and Thomson, 2010; Kessler and Wang, 2012) have recently shown that a body posture that anticipates the direction of mental self-rotation (akin to exocentric perspective-taking) facilitates the transformation, while an incongruent posture delays the process. Importantly, body posture does not affect mental object-rotation (Kessler and Thomson, 2010, Experiment 3). This pattern of results could help in shedding light on the processes engaged by OBE participants during exocentric perspective-taking (i.e., would they show a posture congruency effect or not?). According to their symptomology of perceiving themselves outside their body, we would expect them to engage in self-rotation/exocentric perspective-taking rather than object-rotation whenever possible, making them the highly efficient perspective takers we observed in the current study.

Finally, the current findings also have important bearings on perspective processing in social interactions. Firstly, an intriguing future research question will be if and how OBE participants make use of their efficient perspective-taking skills during social interaction: are they more inclined to adopt another’s perspective in conversation than control participants or are their perspective-taking skills rather confined to visuo-spatial scenarios and completely independent of a social context? We believe that the latter is unlikely in the light of our current findings. In addition and on an anecdotal point, as part of our research programs into anomalous experiences, we have encountered some participants with social phobias/agoraphobias that have reported learning to consciously will and “use” the disembodied viewpoint of the OBE to manage stressful social situations. Here the experience makes the observer feel removed from the direct social context causing stressful reactions.

By moving away from the schematic drawings of the classic OBT task (which have produced mixed results and might not index exocentric perspective-taking) toward more naturalistic photographs of bodies in more varied postures in space, we have enhanced the task’s social dimension especially since these changes have increased the likelihood that motor resonance mechanisms are engaged in order to process difficult body postures (cf. Kessler and Miellet, 2013).

In social interaction, the latter often takes on the form of implicit mimicry, i.e., the so-called “chameleon effect,” which has been shown to enhance pro-social behavior and attitudes (e.g., Chartrand and Bargh, 1999; van Baaren et al., 2009; for review, see Niedenthal et al., 2005). Furthermore, direct effects of posture, posture resonance, and other body-related processes on the speed of egocentric transformations have been recently shown by Kessler and Thomson (2010, especially Experiment 4) and others (e.g., Lenggenhager et al., 2008; Falconer and Mast, 2012; van Elk and Blanke, 2013). Therefore, investigating embodied perspective-taking during realistic social interaction in relation to dissociative traits (e.g., embodied vs. disembodied dissociative experiences) could be a somewhat contra-intuitive, yet highly interesting addition to the field of social cognitive neuroscience.

## CONCLUSION

The present study investigated biases in perspective-taking processes that may be implicated in predisposition to hallucinatory experiences that involve a shift in self-perspective (the OBE). The OBE group were much more efficient at a perspective-taking task relative to a control group – supporting the view that the prevalence of the OBE is associated with biases in perspective-taking ability. In addition, the OBE group displayed significantly more signs of derealization experiences – which we speculate may underlie a propensity to experience ambiguous sensory information from the outside world and may contribute to destabilize the typically coherent sense of self. The current findings also support a fractionating of the unitary notion of dissociation relative to whether embodied or disembodied dissociative experiences are reported. Future studies are planned to investigate the role of both self-perspective inhibition and exocentric perspective-taking underlying these and other related ABEs.

## Conflict of Interest Statement

The authors declare that the research was conducted in the absence of any commercial or financial relationships that could be construed as a potential conflict of interest.

## Footnotes

1 One question on the CDS does ask about feeling “as if” one is outside of the body (question 23) however, this is not regarded as equivalent to the more direct question of actually “perceiving” the world from an external point of view. In addition, further questioning with DPD patients reveals this is rarely, if ever, defined as an OBE by the patient.

2 All removed participants were from the control group. Exit questioning revealed that some described the experiment as “too hard” so they did not engage fully with the experiment and others that they were confused about the instructions.

3 This suggestion assumes that the term “disembodied” be interpreted more literally. By this taxonomy, one cannot have an egocentric disembodied experience, but one can have an egocentric dissociative experience.

4 Importantly, “*embodied dissociation*” and “*disembodied dissociation*” are not being argued to be absolute – more so that these processes likely co-exist as relative biases – where one dictates and defines anomalous conscious experience at any given time. For example, while primarily disembodied, OBEs can consist of a minor co-awareness of the physical self. Nonetheless, disembodied processes dominate the phenomenology and realism of the experience. Exploring the presence, degree, and interplay of such biases, across different OBEs and associated experiences, will be an exciting avenue for future research.
